# A New Method for Non-Invasive Estimation of Human Muscle Fiber Type Composition

**DOI:** 10.1371/journal.pone.0021956

**Published:** 2011-07-07

**Authors:** Audrey Baguet, Inge Everaert, Peter Hespel, Mirko Petrovic, Eric Achten, Wim Derave

**Affiliations:** 1 Department of Movement and Sport Sciences, Ghent University, Ghent, Belgium; 2 Research Center for Exercise and Health, K.U. Leuven, Leuven, Belgium; 3 Department of Internal Medicine, Ghent University, Ghent, Belgium; 4 Department of Radiology, Ghent Institute for Functional and Metabolic Imaging (GIfMI), Ghent University, Ghent, Belgium; Institut Pluridisciplinaire Hubert Curien, France

## Abstract

**Background:**

It has been established that excellence in sports with short and long exercise duration requires a high proportion of fast-twitch (FT) or type-II fibers and slow-twitch (ST) or type-I fibers, respectively. Until today, the muscle biopsy method is still accepted as gold standard to measure muscle fiber type composition. Because of its invasive nature and high sampling variance, it would be useful to develop a non-invasive alternative.

**Methodology:**

Eighty-three control subjects, 15 talented young track-and-field athletes, 51 elite athletes and 14 ex-athletes volunteered to participate in the current study. The carnosine content of all 163 subjects was measured in the gastrocnemius muscle by proton magnetic resonance spectroscopy (^1^H-MRS). Muscle biopsies for fiber typing were taken from 12 untrained males.

**Principal Findings:**

A significant positive correlation was found between muscle carnosine, measured by ^1^H-MRS, and percentage area occupied by type II fibers. Explosive athletes had ∼30% higher carnosine levels compared to a reference population, whereas it was ∼20% lower than normal in typical endurance athletes. Similar results were found in young talents and ex-athletes. When active elite runners were ranked according to their best running distance, a negative sigmoidal curve was found between logarithm of running distance and muscle carnosine.

**Conclusions:**

Muscle carnosine content shows a good reflection of the disciplines of elite track-and-field athletes and is able to distinguish between individual track running distances. The differences between endurance and sprint muscle types is also observed in young talents and former athletes, suggesting this characteristic is genetically determined and can be applied in early talent identification. This quick method provides a valid alternative for the muscle biopsy method. In addition, this technique may also contribute to the diagnosis and monitoring of many conditions and diseases that are characterized by an altered muscle fiber type composition.

## Introduction

In mammals, including humans, skeletal muscle fibers exist in two main categories, the fatigue-resistant slow-twitch (ST) or type-I fibers, and the fatigue-sensitive fast-twitch (FT) or type-II fibers [1]. Classical papers from the 70s [2], [3] established that excellence in sports with short and long exercise duration requires a high proportion of FT and ST muscle fibers, respectively. There is an ongoing nature/nurture debate whether a fiber can modify into another type in humans, but the effect of specific types of exercise training on the transition between ST and FT fiber population is probably limited [4]. Therefore, measurement of the muscle fiber type composition can be a tool for talent identification and for defining an athlete's optimal exercise duration in track-and-field as well as many other sports. Moreover, some conditions (space-flight-induced muscle atrophy [5]) and diseases, such as type-II diabetes [6], neurodegenerative disorders and sarcopenia [7], spinal cord injury [8], cancer cachexia [9], chronic obstructive pulmonary disease (COPD) [10] are characterized by an altered muscle fiber type composition. Because of the invasive nature and high sampling variance of the muscle biopsy method, a non-invasive alternative to measure muscle fiber type composition would be useful.

Several attempts have previously been made to determine muscle fiber type composition in a non-invasive way in resting muscles. Researchers used magnetic resonance imaging (MRI), to investigate the relation between T1 and T2 relaxation times on the one hand and the percentage type-I fibers on the other hand [11]–[13].The results are contradictory and suggest that MRI is not useful for the determination of muscle fiber type composition. Phosphorus magnetic resonance spectroscopy (^31^P-MRS) has also been used to measure metabolites that differ between type-I and type-II muscle fibers. Bernus and colleagues reported that inorganic phosphate (Pi) is lower in endurance athletes, while they are characterized by higher levels of phosphocreatine (PCr), compared to sprinters [14]. However, also the use of ^31^P-MRS for non-invasive fiber typing did result in equivocal conclusions, probably because the phosphorous metabolite concentrations are not strongly different between fiber types.

Recently, ^1^H-MRS has been used to measure muscle metabolites, such as intra- (IMCL) and extra-myocellular lipids (EMCL), trimethylamonium (TMA) and carnosine. Two important requirements to use a metabolite for the determination of muscle fiber composition, are that the concentrations are markedly different between type-I and II fibers, and in addition are largely independent of extrinsic factors such as diet and training. With this in mind, carnosine seemed to be a good candidate.

The dipeptide carnosine is present in high concentrations in muscle and is a relatively stable characteristic of human skeletal muscle (approximately 10% variation over a 3-month period [15]). Only long-term vegetarianism [16] or high-dose beta-alanine supplementation for several weeks can change the muscle carnosine content [17]. Short-term exercise training would probably have little or no impact on muscle carnosine levels [18]–[21]. However, muscle fiber type is a major determinant of carnosine levels. FT fibers contain twice as much carnosine as the ST fibers [22], [23]. Consequently, marathon runners seem to have low muscle carnosine content [24]. In untrained subjects, moderately positive correlations have been found between FT fiber proportion and carnosine content, using muscle biopsies [25], [26].

The aim of the current study was to develop a new and non-invasive estimation method of fiber type composition in human muscles, based on proton magnetic resonance spectroscopy (^1^H-MRS) measurement of muscle carnosine content, a typical FT metabolite.

## Materials and Methods

### Subjects

**Figure 1:**
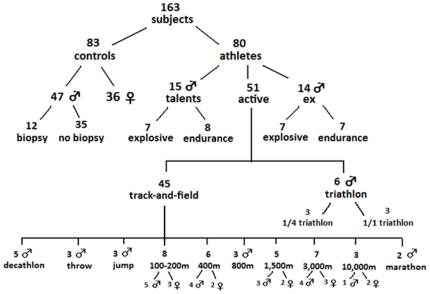
Overview of subject population. Numbers of subjects per group and by gender is shown. Muscle carnosine content is measured of all 163 subjects and muscle biopsies were taken from 12 not specifically trained males.

A total of 163 subjects volunteered to participate in this cross-sectional study. As can be seen on figure 1, the study population consists of 83 not specifically trained controls (47 males and 36 females) and 80 athletes (68 males and 12 females). The reference population was physically active, but not involved in competitive sport or organized training. The athletes were divided in 3 subgroups: 1) 15 talented young male track-and-field athletes, 2) 51 active elite athletes (39 males, 12 females) and 3) 14 male ex-athletes. All active and ex-athletes were or had been competing at international level. Nineteen of them won a medal at European or World Championships or the Olympics. The active elite athletes were recruited from triathlon (n = 6) and track-and-field (n = 45). The 45 track-and-field athletes were assigned to 1 of the following disciplines; 100–200 m, 400 m, 800 m, 1,500 m, 3,000-marathon, jump, throw, decathlon, using the IAAF scoring tables of athletics [27]. The young talented athletes (n = 15) and the former athletes (n = 14) were divided into an explosive and endurance group. None of the subjects was vegetarian or had taken beta-alanine in the 3 months before the start of the study. All subjects gave their written informed consent and the study was approved by the local ethics committee (Ghent University Hospital, Belgium).

### Muscle fiber typing

Muscle biopsies were taken at rest from the gastrocnemius of 12 males of the reference population, with a 14 Gauge true-cut biopsy needle (Bard Magnum Biopsy gun; Bard, Inc., New Jersey, USA). Under ultrasonographic guidance (Ultrasonography Pro Sound SSD-5000, ALOKA Co., Ltd., Tokyo, Japan. with probe UST-5545, frequency 5–13MHZ), 3 muscle samples were taken following local anesthesia (lidocaine 1%, Linisol®). The samples were frozen in nitrogen-cooled isopentane and embedded in Tissue-Tek for immunohistochemical analysis. Muscle cryosections were immunohistochemically stained for myosin heavy chain isoforms and analyzed as previously described [28].

### Muscle carnosine content

The carnosine content of the gastrocnemius muscle was measured by proton magnetic resonance spectroscopy (^1^H-MRS) in all 163 subjects, as previously described [29]. The subjects were lying in supine position on their back and the lower leg was fixed in a holder with the angle of the ankle at 20° plantar flexion. All the MRS measurements were performed on a 3 Tesla whole body MRI scanner (Siemens Trio, Erlangen) equipped with a spherical knee-coil. Single voxel point-resolved spectroscopy (PRESS) sequence with the following parameters was used: repetition time (TR) = 2.000 ms, echo time (TE) = 30 ms, number of excitations = 128, 1.024 data points, spectral bandwidth of 1.200 Hz, and a total acquisition time of 4.24 min. The average voxel size was 40 mm×12 mm×28 mm and the line width of the water signal was on average 25.7 Hz, following shimming procedures. The absolute carnosine content (in millimolar; mM) was calculated as described before by Baguet et al 2010 [29], using the following formula:
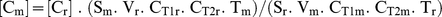
[C_m_]: carnosine concentration in vivo; [C_r_]: carnosine concentration of the external reference phantom (20 mM); S_m_ and S_r_: estimated signal peak areas of the muscle and reference phantom; V_m_ and V_r_: voxel volumes of the muscle and reference phantom; C_T1m_, C_T2m_, C_T1r_ and C_T2r_: correction factors for the T1 and T2 relaxation times in the muscle and in the reference phantom; T_m_ and T_r_: temperatures in the muscle and in the reference phantom.

Previous studies showed a variation coefficient of gastrocnemius carnosine content over a 6-week period of 11.9% in untrained [15] and 13.2% in trained subjects [17], [29]. In the current study, the athletes' muscle carnosine content was measured in both right and left leg to reduce the variation. Given the higher carnosine concentrations in men compared with women [30], Z-scores are used, instead of absolute values, when men and women are displayed in the same figure. Z-scores for men and women were calculated using mean and standard deviation of the male and female reference population, respectively.

### Statistics

**Figure 2:**
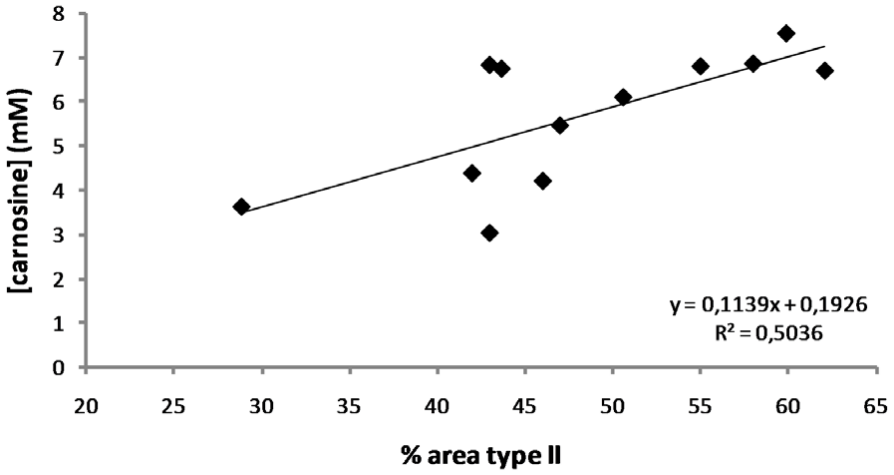
Correlation between muscle carnosine content and percentage area occupied by type-II fibers in 12 untrained subjects. The X-axis displays the percentage of the total area occupied by type-II fibers. The muscle carnosine content in millimolar (mM) is shown on the Y-axis. A significant (p = 0.009 and r = 0.714) positive correlation between muscle carnosine content and percentage area occupied by type-II fibers is demonstrated.

The correlation in figure 2 was evaluated by a Pearson correlation. Independent sample T-tests were used to compare the muscle carnosine content between explosive and endurance athletes in talents, elites and ex-athletes (SPSS statistical software, SPSS 17.0, Chicago, IL). Values are presented as means ± SD and significance was assumed at p≤0.05. The sigmoidal curve was designed with SigmaPlot 11 (Systat Software Inc.).

## Results

### Relationship between muscle carnosine content and fast-twitch fiber area

In the control population, muscle carnosine ranged from 1.68 mM to 7.85 mM in males and from 1.82 mM to 6.25 mM in females. In the 12 untrained subjects, the biopsy-determined percentage FT fiber area ranged between 29 and 62%. Figure 2 shows a strong positive correlation (p = 0.009 and r = 0.714) between ^1^H-MRS-based carnosine concentration and the percentage FT fiber area in gastrocnemius muscle.

### Muscle carnosine content in active elite athletes

**Figure 3:**
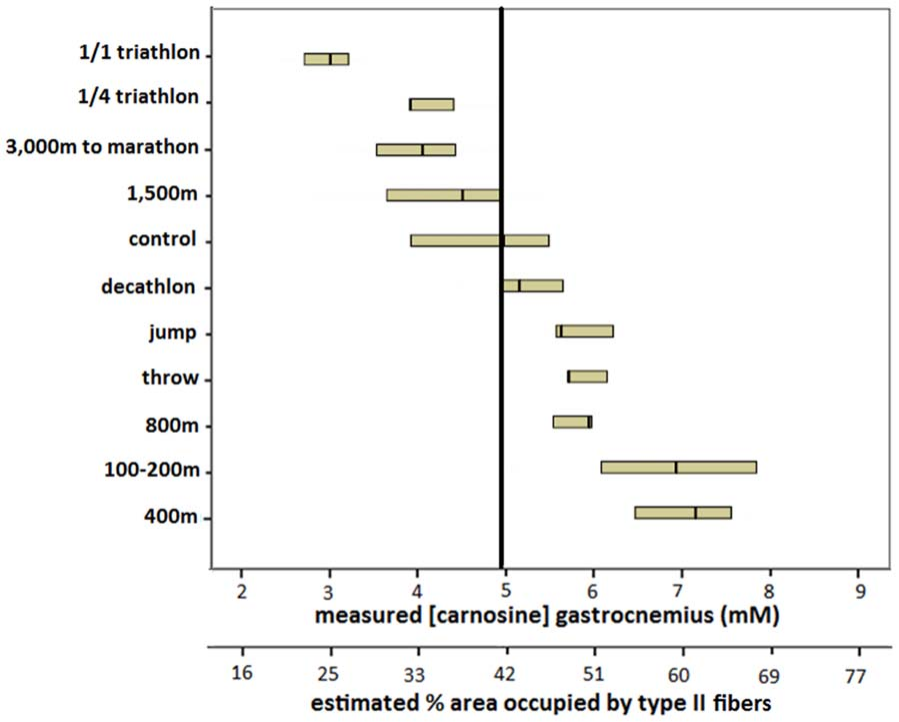
Carnosine content of gastrocnemius muscle in track-and field and triathletes compared to an untrained male control population. Muscle carnosine content (mM) in various small groups of male elite athletes (n = 39) and in a male control population (n = 47) ranked from low to high. Numbers per group are demonstrated in figure 1. The primary X-axis shows the measured carnosine content, while the secondary X-axis displays the estimated percentage area occupied by type II fibers (derived from figure 2). The vertical line represents the median of the male control population. Medians (small vertical lines) and first and third quartile are shown by group.

**Figure 4:**
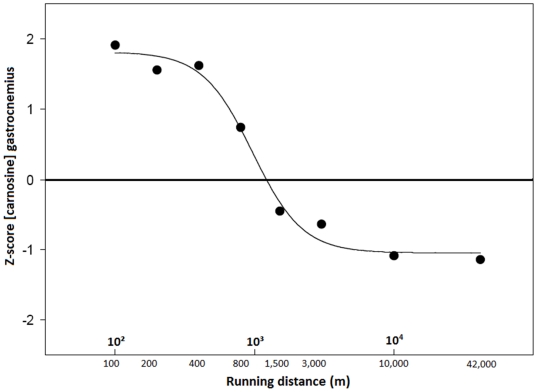
Carnosine content of gastrocnemius muscle in male and female active elite runners, according to their best running distance. All male (n = 22) and female (n = 12) elite runners were ranked according to their best running distance (using the IAAF scoring tables of athletics). The X-axis displays running distance (m) in a logarithmic fashion and the Y-axis shows the carnosine content, expressed in Z-scores. A Y-value of zero corresponds to the average of the male/female reference population. The median Z-score per group is shown and the best sigmoidal fit is presented.

In a Belgian male elite athlete population, the muscle carnosine content, measured by ^1^H-MRS, was ∼30% higher (p<0.001) in explosive athletes (6.58±0.92 mM), such as sprint runners, than in a reference population (4.94±1.43 mM), whereas this was ∼20% lower (p = 0.11) than normal in typical endurance athletes (3.75±0.74 mM), such as 3,000 m-marathon runners and triathletes (figure 3). Sprinters (100–400 m) had on average 1.9-fold higher carnosine content than marathon runners and triathletes (p<0.001). All 100 to 400 m runners had a higher muscle carnosine content than the population mean and 100% of triathletes and marathon runners had a lower carnosine concentration than the average of the reference population. Athletes competing in disciplines requiring both sprint and endurance capacities, such as decathletes, showed intermediate carnosine levels (5.32±0.72 mM). We observed the same pattern in female athletes (data not shown), although absolute carnosine concentrations were consistently lower in women than in men, in agreement with previous reports [30]. Furthermore, figure 4 displays all male and female active elite runners (n = 34) ranked according to their best running distance. Interestingly, a negative sigmoidal (R^2^ = 0.9874), rather than linear relation was found between the logarithm of the best running distance and muscle carnosine content, expressed in Z-scores.

### Muscle carnosine content in young and former athletes

**Figure 5:**
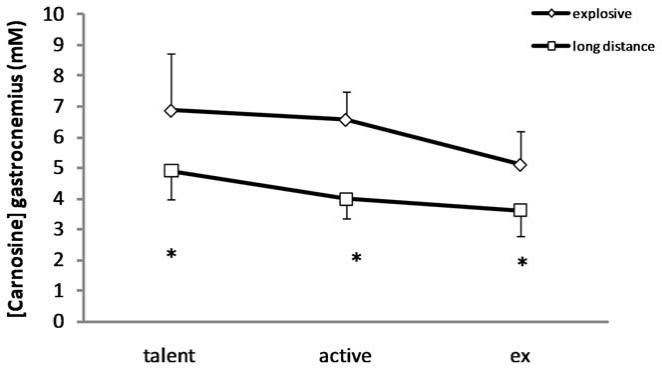
Comparison of the gastrocnemius carnosine content in male young talented athletes (n = 15), active elite athletes (n = 19) and ex-athletes (n = 14). The Y-axis demonstrates the carnosine content in millimolar (mM). Diamonds represent explosive athletes, including sprinters and jumpers (talents n = 7; active n = 12 ; ex n = 7) and squares endurance runners (talents n = 8; active n = 7; ex n = 7). Data are shown as means ± standard deviation. * Different from explosive athletes (p≤0.05).

Noteworthy is that the muscle carnosine concentration remained significantly different between ex-sprinters (n = 7) and ex-endurance athletes (n = 7) (5.11±1.07 vs. 3.61±0.81 mM; p = 0.012), who had discontinued training for many years already. Moreover, a similar difference (p = 0.019) was observed in young talents between explosive (n = 7) (6.88±1.83 mM) and endurance (n = 8) (4.90±0.93 mM) athletes (figure 5).

## Discussion

In order to develop a new non-invasive method to estimate muscle fiber composition in humans, we measured the muscle carnosine content of 80 Belgian track-and-field athletes and 83 not specifically trained controls, using ^1^H-MRS. Athletes excelling in 100 m to 400 m all exhibit a high carnosine level, likely because these distances require a similar high percentage of fast-type muscle fibers [3], [31], whereas 1,500–3,000 m and especially 10,000 m to marathon runners have low carnosine content, likely because these distances require a high percentage of slow-type muscle fiber [3], [31]. In fact, the 800 m runners show least resemblance with any other distance as their muscle carnosine content lies in between those of sprint and endurance athletes and is situated on the steepest part of the sigmoidal curve (the midpoint of the curve lies at ∼1,000 m). We also looked at other proton MRS metabolites, such as creatine and IMCL, but they did not correlate with athletic disciplines (data not shown).

It could be opposed that the higher carnosine content in the sprinters is perhaps an acute response to intensive training, rather than a reflection of a predominantly fast muscle fiber type composition. However, the muscle carnosine concentration remained significantly different (5.11±1.07 vs. 3.61±0.81 mM; p = 0.012) between ex-sprinters and ex-endurance athletes, who had discontinued training for many years already. Moreover, a similar significant (p = 0.019) difference was observed in young talents (14–18 years old) between endurance and explosive athletes. As these young athletes are still at the start of their career and as the accumulated training history is several thousands of hours less than their elite adult colleagues, this suggests that the muscle carnosine content is probably largely genetically determined (figure 5), as is the muscle fiber type composition. Therefore, we believe that this new method may prove useful in the early identification of talents in sports where muscle fiber type composition is a determining factor.

Van Damme et al. [32] explored the performance constraints in elite decathletes and concluded that performances on different subdisciplines, like 100 m and 1,500 m, correlate negatively, partly because of the conflicting muscle fiber type requirements. Moreover, excellence in a particular discipline (specialist) is detrimental for overall decathlon performance (generalist). Our findings in muscle seem to agree with both of these points. With respect to muscle carnosine, disciplines like 100 m and 1,500 m indeed have antagonistic requirements (figure 4). Additionally, the 5 elite decathletes we measured (figure 3) had intermediate carnosine levels within a relatively narrow range, suggesting that they were all generalists, rather than specialists.

This novel method provides a non-invasive estimation of human muscle fiber type composition. An important advantage of this method is that it does not induce damage to the muscle, in contrast to the biopsy method, which makes it 1) very useful beyond the laboratory environment, 2) infinitely repeatable and 3) applicable to special populations, such as elite athletes and children. A second disadvantage of the biopsy method is the representativity. When a biopsy is taken, typically the fiber typing is done on a tissue sample representing less than 0.01% of the total muscle mass [33] and containing only a couple of hundreds of fibers of even less motor units. Therefore, a single biopsy is a poor estimator of the whole muscle fiber type distribution [34], [35] and multiple biopsies are required to adequately estimate the muscle fiber type distribution [36], [37]. The current NMR-based method typically samples 10–15 ml of muscle, including both superficial and deep parts of the muscle and representing approximately 5% of the entire muscle. MRS-based carnosine quantification has a relatively good repeatability in untrained [15] and trained [17], [29] humans. A third advantage compared to the biopsy method, is that the technique is not labor-intensive and the scanning and analysis is performed in 30 minutes. The MRS-based technique therefore tackles most of the disadvantages of the biopsy method (invasive – low representativity – labor intensive) that have kept the technique from evolving from a research tool towards a routine screening method for predicting athletic success [38].

A weakness, however, of the current method is that it is based on indirect estimation through quantification of a single metabolite carnosine, which is a typical metabolite of FT fibers. Certain nutritional interventions such as chronic vegetarianism [16] or beta-alanine supplementation [17], [39] can influence the muscle carnosine content without altering the fiber type composition, which will disrupt the relationship between muscle fiber type composition and carnosine content. These nutritional states were therefore treated as exclusion criteria in the present study. Also the applicability in certain pathologies should be evaluated in order to exclude a metabolic effect of the disease on the carnosine content itself.

In conclusion, ^1^H-MRS based carnosine quantification is a valuable non-invasive approach to estimate muscle fiber type composition, based on the close level of agreement with the performance characteristics of various small groups of elite athletes. This fast and easy method may have useful applications in talent identification and sport discipline (re)orientation as well as in the muscle evaluation of various pathological conditions.
